# SMARCB1 Acts as a Quiescent Gatekeeper for Cell Cycle and Immune Response in Human Cells

**DOI:** 10.3390/ijms21113969

**Published:** 2020-06-01

**Authors:** Sung Kyung Choi, Myoung Jun Kim, Jueng Soo You

**Affiliations:** Department of Biochemistry, School of Medicine, Research Institute of Medical Science, Konkuk University, Seoul 05029, Korea; cskyung919191@daum.net (S.K.C.); wns798@naver.com (M.J.K.)

**Keywords:** SMARCB1, interleukin 6, SWI-SNF, cell cycle, immune response

## Abstract

Switch/sucrose non-fermentable (SWI/SNF)-related matrix-associated actin-dependent regulator of chromatin (SMARC) subfamily B member 1 (SMARCB1) is a core subunit of the switch/sucrose non-fermentable (SWI/SNF) complex, one of the adenosine triphosphate (ATP)-dependent chromatin remodeler complexes. The unique role of SMARCB1 has been reported in various cellular contexts. Here, we focused on the general role of the ubiquitous expression of SMARCB1 in a normal cell state. We selected ARPE19 (human primary retinal pigment epithelium) and IMR90 (from human fetal lung fibroblasts) cell lines as they have completely different contexts. Furthermore, although these cell lines have been immortalized, they are relatively close to normal human cells. The loss of SMARCB1 in ARPE19 and IMR90 cells reduced cell cycle progression via the upregulation of P21. Transcriptome analysis followed by SMARCB1 knockdown in both cell lines revealed that SMARCB1 was not only involved in cell maintenance but also conferred immunomodulation. Of note, SMARCB1 bound to interleukin (IL) 6 promoter in a steady state and dissociated in an active immune response state, suggesting that SMARCB1 was a direct repressor of IL6, which was further confirmed via loss- and gain-of-function studies. Taken together, we demonstrated that SMARCB1 is a critical gatekeeper molecule of the cell cycle and immune response.

## 1. Introduction

Chromatin-remodeling, which causes changes in chromatin structure, is essential for regulating the potential for gene expression [1]. Chromatin-regulating proteins modify the chromatin structure through the dynamic modification of DNA or histones and remodel DNA–histone interaction [2]. The altered chromatin structure activates or represses gene transcription [2,3]. Adenosine triphosphate (ATP)-dependent chromatin-remodeling enzymes use energy from ATP hydrolysis and are one of the main groups of chromatin-regulating proteins [4]. This group is highly conserved and includes switch/sucrose non-fermentable (SWI/SNF), imitation switch (ISWI), nucleosome remodeling deacetylase (NURD)/Mi-2/chromodomain helicase DNA-binding protein (CHD), and INO80, which are well-known chromatin remodelers that form multi-subunit complexes [5,6]. In particular, SWI/SNF comprises approximately 15 subunits, and each subunit change is associated with a specific function [7]. The ATPase catalytic subunits of SWI/SNF, brahma homolog (BRM), or brahma-related gene 1 (BRG1), can act as activators or repressors of transcription, and they are either mutually exclusive or compensate for each other [8,9,10]. However, SWI/SNF-related matrix-associated actin-dependent regulator of chromatin (SMARC) subfamily B member 1 (SMARCB1; SWI/SNF chromatin-remodeling complex subunit SNF5, BRG1-associated factor (BAF) 47, and integrase interactor 1 (INI1)), one of the core subunits of SWI/SNF, remains unchanged and is ubiquitously expressed [11]. SMARCB1 has been extensively studied as a critical player in cell state regulation. One of its well-known roles is as a tumor suppressor gene [12], and it is genetically mutated/inactivated over a wide spectrum of cancers, including rhabdoid, brain, kidney, and other tissue tumors [13,14,15]. One of the SMARCB1-related oncogenic pathways is cell cycle regulation. SMARCB1 transcriptionally regulates cell cycle-related genes. It represses cyclin D1; inactivates retinoblastoma (RB); represses E2 promoter binding factor (E2F) target genes, such as cyclin A, E2F1, and CDC6; and represses c-MYC through direct interaction, resulting in cell cycle arrest [16,17,18]. SMARCB1 performs other specific functions by modulating nucleosome positioning and silencing octamer-binding transcription factor 4 (OCT4), which is crucial for the cellular differentiation of embryonic stem cells. SMARCB1 maintains a balance between pluripotency and differentiation by controlling the OCT4 target gene expression [19]. However, considering the ubiquitous gene expression of SMARCB1, it is crucial to elucidate its general role in a normal state.

Normal cells have several experimental limitations, such as limited proliferative potential caused by senescence or a finite number of cell divisions [20]. To overcome this limitation, researchers have manipulated cells to make them immortal by overriding the cell cycle, maintaining telomere length, and avoiding cellular senescence [21]. We performed our experiments with two representative immortalized human cell lines, ARPE19 and IMR90. ARPE19 evolved from human primary RPE (retinal pigment epithelium) cells that spontaneously immortalized [22], and this cell line is extremely useful for studying retinoid metabolism or RPE-specific gene expression [23]. IMR90 cells are human telomerase reverse transcriptase (H-Tert)-immortalized human fibroblasts derived from human fetal lung fibroblasts and are immortalized [24]. IMR90 has been used in many studies as representative fibroblasts.

The immune response is a defense mechanism to maintain homeostasis in cases of infection, injury, and malfunction [25]. It is the basic base of all biological responses, and balancing between a pathogenic and protective immune response is critical not only for immune cells but also for all other types of cells to maintain health [26]. Inflammation cascades are extremely complex and multidimensional processes coordinated by cytokines and immune-signaling molecules [27]. The modification of chromatin structure is necessary to regulate the expression levels of these cytokines and immune-signaling molecules. Previous investigations have revealed the transcriptional regulation of cytokines and immune-signaling molecules by chromatin remodelers [28,29]. SMARCE1 (also known as BAF57) represses CD4 transcription through facilitating and remodeling H1-containing chromatin at the CD4 silencer during T-cell development [28]. Protein polybromo-1 (PBRM1 also known as BAF180) directly binds to regulatory elements of interleukin (IL) 10 to negatively regulate IL10 transcription in T-helper 2 (Th2) cells [29]. Furthermore, the BAF complex is crucial for the induction of interferon (IFN) signaling and the transcription of IFN-stimulated genes [13,30,31]. The BAF complex is required to maintain the open conformation of the IFN target promoter [32]. While these studies have presented the relationship between the SWI/SNF complex and the immune response, further research is necessary to elucidate the molecular network between two.

This study investigated the general role of SMARCB1 in immortalized human cell lines. The loss of SMARCB1 induced cell cycle arrest. Based on transcriptome analysis, we analyzed commonly up- and downregulated genes by SMARCB1 knockdown in ARPE19 and IMR90 cell lines. The genes coregulated by SMARCB1 were associated with inflammation-related phenomena, particularly IL6 signaling via IL6 transcription regulation. SMARCB1 was associated with cell cycle regulation and immune response for cellular maintenance in normal cells.

## 2. Results

### 2.1. SMARCB1 Controls Cell Cycle

**Figure 1:**
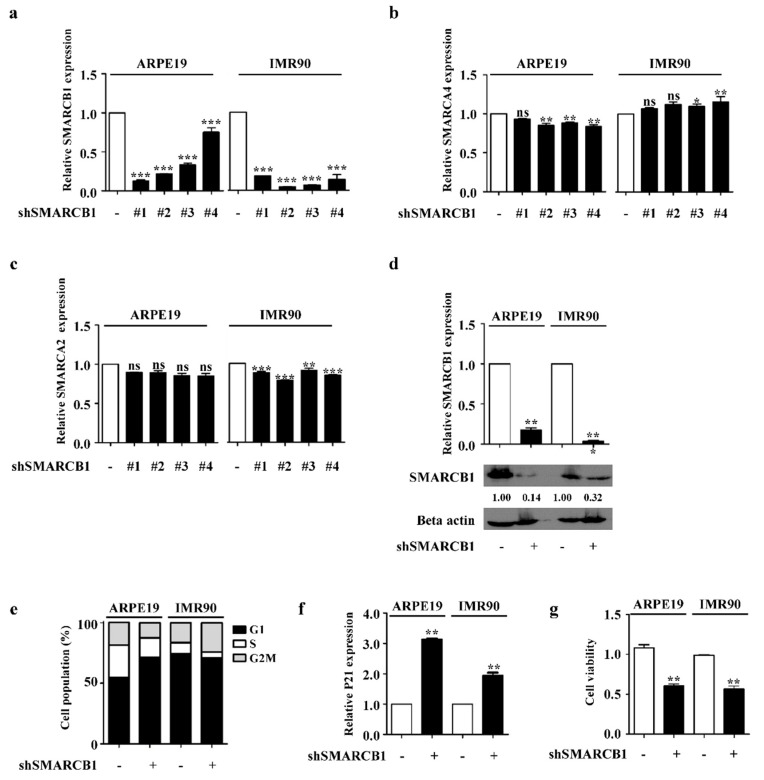
SMARCB1 controls cell cycle progression in human cell lines. (**a**) mRNA level of SMARCB1 determined by real-time PCR in ARPE19 and IMR90 (#1–4). We performed three independent experiments and analyzed statistically (mean +/− S.E.M, Statistical significance *** *p* < 0.001 vs. shControl). (**b**) mRNA level of SMARCA4 determined by real-time PCR in ARPE19 and IMR90 (#1–4). We performed three independent experiments and analyzed statistically (mean +/− S.E.M, ns: non significant, Statistical significance * *p* < 0.05, ** *p* < 0.01 vs. shControl). (**c**) mRNA level of SMARCA2 determined by real-time PCR in ARPE19 and IMR90 (#1–4). We performed three independent experiments and analyzed statistically (mean +/− S.E.M, ns: non significant, Statistical significance ** *p* < 0.01, *** *p* < 0.001 vs. shControl). (**d**) mRNA level of SMARCB1 (top) and protein level of SMARCB1 (bottom) determined by real-time PCR and Western blot analyses in ARPE19 and IMR90. We performed three independent experiments and analyzed statistically (mean +/− S.E.M, Statistical significance ** *p* < 0.01 vs. shControl). (**e**) Cell cycle in SMARCB1 knockdown ARPE19 and IMR90 determined by PI staining. (**f**) mRNA level of P21 determined by real-time PCR in ARPE19 and IMR90. We performed three independent experiments and analyzed statistically (mean +/− S.E.M, Statistical significance ** *p* < 0.01 vs. shControl). (**g**) Cell viability analysis in SMARCB1 knockdown ARPE19 and IMR90 determined by MTT (3-(4,5-dimethylthiazol-2-yl)-2,5-diphenyltetrazolium bromide) assay. We performed three independent experiments and analyzed statistically. (mean +/− S.E.M, Statistical significance ** *p* < 0.01 vs. shControl).

To determine the role of SMARCB1 in normal human cells, we generated stable SMARCB1-knockdown cell lines using short hairpin (sh)RNA in ARPE19 and IMR90 cells. After comparing the knockdown efficiency of four shSMARCB1 viruses, we selected cell line #2, which showed a high knockdown efficiency in both ARPE19 and IMR90 cells (Figure 1a). Although the difference was small, we found that SMARCB1 knockdown did affect the expression of other subunits, SMARCA4 and SMARCB2. However, the expression pattern was not correlated in ARPE19 and IMR90 (Figure 1b,c). We confirmed the loss of SMARCB1 by reverse transcription (RT)-polymerase chain reaction (PCR) and Western blot analysis in both cell lines at the same time (Figure 1d). SMARCB1 knockdown induced cell cycle arrest, upregulation of P21 expression, and reduced cell viability in both cell lines (Figure 1e–g) The cell cycle phase distribution is different in SMARCB1 knockdown; the G1 phase was increased in ARPE19 and the G2M phase was increased in IMR90. We thought that the different cellular context and culture environment of the two cell lines might have influenced the difference in this result. We suggest that SMARCB1 played a key role in cell cycle progression in human cell lines.

### 2.2. SMARCB1 Modulates the Transcriptome in Cellular Maintenance and Immune Response

**Figure 2:**
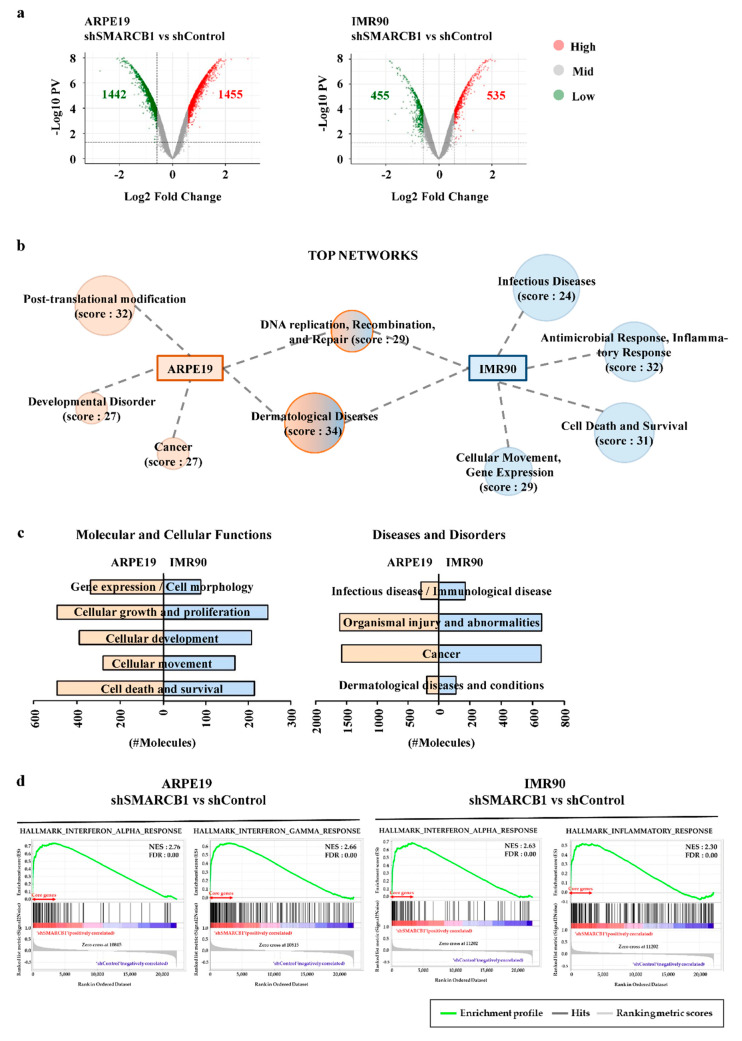
SMARCB1 modulates similar transcriptome in ARPE19 and IMR90. (**a**) The volcano plot of statistical significance against 1.5 fold change in ARPE19 and IMR90. (**b**) Highly ranked and common networks of SMARCB1 knockdown ARPE19 and IMR90 based on ingenuity pathway analysis (IPA). The score is based on a *p*-value calculation, which calculates the likelihood that the network-eligible molecules that are part of a network are found therein by random chance alone. (**c**) The most significant and common diseases and bio functions of SMARCB1 knockdown ARPE19 and IMR90. The number of molecules (genes) that are associated with each function. (**d**) Immune response-associated gene set enrichment analysis (GSEA) of SMARCB1 knockdown ARPE19 and IMR90.

We used microarrays to investigate the similarities and differences in the SMARCB1-dependent transcript landscape in each cell line. Following SMARCB1 knockdown, 1455 genes were upregulated and 1442 genes were downregulated in ARPE19 cells, and 535 genes were upregulated and 455 genes were downregulated in IMR90 cells (fold change ≥ 1.5, *p*-value < 0.05). Considering that the number of altered genes (up or down) was similar, SMARCB1 might play a role in transcription activation and repression in both cell lines [33,34,35] (Figure 2a). To predict the downstream pathway regulated by SMARCB1, we performed ingenuity pathway analysis (IPA). Although posttranslational modification revealed a unique network in ARPE19 cells, both cell lines shared top networks, molecular and cellular functions, and diseases (Figure 2b,c). In particular, as shown in the general canonical pathway analysis, cell death, survival, and involvement in cell growth and proliferation explained why knockdown of SMARCB1 caused cell cycle arrest (Figure 2c). Unexpectedly, we found that infectious and immunological disease had high importance in both cell lines (Figure 2c), presuming that SMARCB1 is functional in immune reactions in both cell lines. Furthermore, gene set enrichment analysis (GSEA) results also supported the idea that the gene sets associated with IFNα response and inflammatory response were upregulated in SMARCB1-knockdown cells (Figure 2d). These findings suggested that SMARCB1 could modulate the transcriptome to regulate cellular maintenance and immune response in vastly different contexts.

### 2.3. SMARCB1 Loss Activates the Immune Response Gene Set and Represses the Cell Maintenance Gene Set

**Figure 3:**
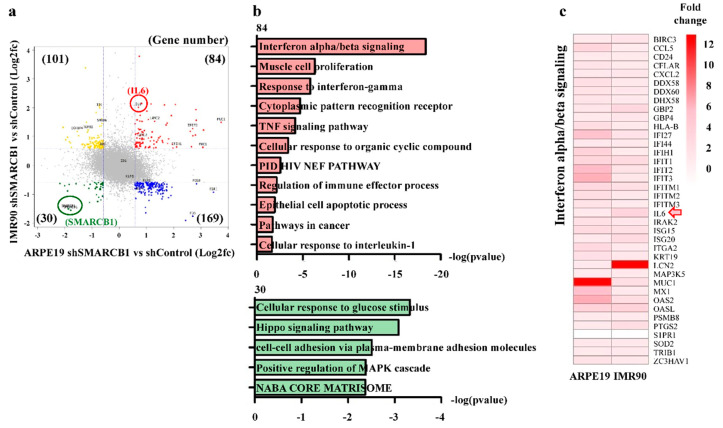
SMARCB1 regulates immune response gene set and cell maintenance gene set. (**a**) Correlations and scatter plots of gene expression in SMARCB1 knockdown ARPE19 and IMR90. (**b**) Gene ontology (GO) analysis of commonly upregulated or downregulated genes in SMARCB1 knockdown ARPE19 and IMR90. (**c**) Heatmap showing expression of interferon alpha/beta signaling genes in SMARCB1 knockdown ARPE19 and IMR90 (arrow: labeling common SMARCB1 target IL6).

Although both ARPE19 and IMR90 cell lines are considered normal, their context is very different. ARPE19 is an epithelial cell line, and IMR90 is a mesenchymal cell line [22,23,24]. To determine the common downstream target of SMARCB1 in different contexts, we analyzed quadrant scatter plots based on up- or downregulated genes by SMARCB1 knockdown in ARPE19 and IMR90 cells. The SMARCB1 knockdown cells had 84 upregulated genes and 30 downregulated genes in common, including SMARCB1 (Figure 3a). Next, we performed a DAVID analysis (https://david.ncifcrf.gov/) and found that the commonly upregulated genes (*n* = 84) were mainly involved in immune-related phenomena, such as IFNα/ꞵ signaling, IFNγ response, and the tumor necrosis factor (TNF) signaling pathway (Figure 3b), further validating our IPA and GSEA results (Figure 2). By contrast, the commonly downregulated genes (*n* = 30) were associated with cell maintenance and proliferation, such as the cellular response to glucose stimulus and the positive regulation of the mitogen-activated protein kinase (MAPK) cascade [36,37] (Figure 3b). Multifunctional cytokine IL6, which regulates the inflammatory response and immune reaction [38], was identified in the IFNα/ꞵ signaling gene set, which was the most significantly upregulated gene set (Figure 3c). Taken together, we hypothesized that SMARCB1 could modulate the immune response through IL6.

### 2.4. SMARCB1 Directly Regulates IL6 as a Transcriptional Repressor

**Figure 4:**
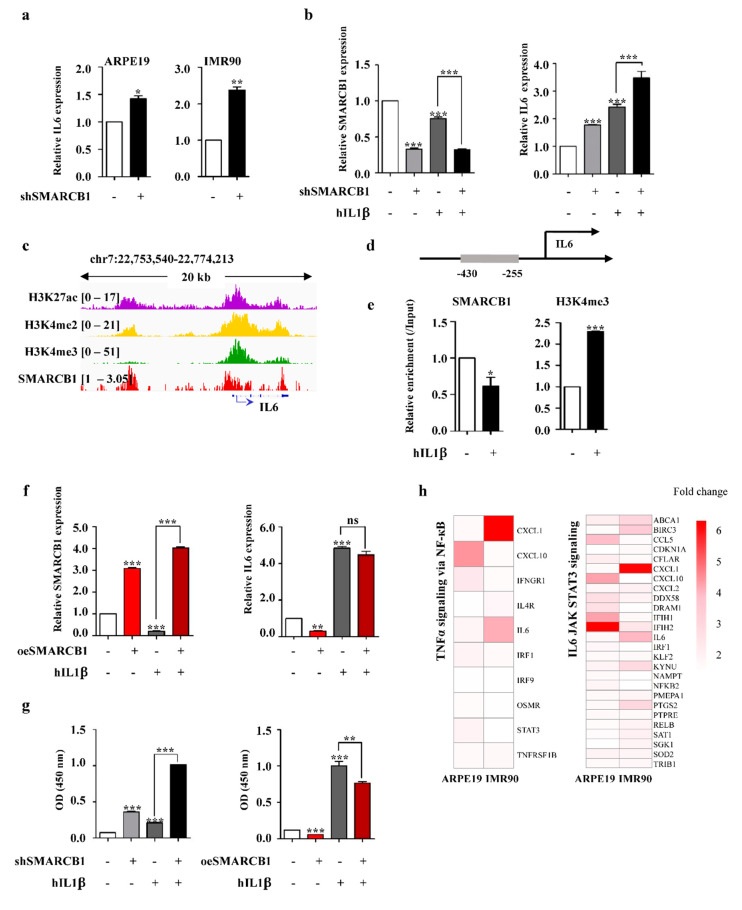
SMARCB1 directly regulates IL6. (**a**) mRNA level of IL6 determined by real-time PCR in SMARCB1 knockdown ARPE19 and IMR90. We performed three independent experiments and analyzed statistically (mean +/− S.E.M, Statistical significance * *p* < 0.05, ** *p* < 0.01 vs. shControl). (**b**) mRNA level of SMARCB1 and IL6 determined by real-time PCR in SMARCB1 knockdown and/or hIL1β treated ARPE19. We performed three independent experiments and analyzed statistically (mean +/− S.E.M, Statistical significance *** *p* < 0.001 vs. shControl). (**c**) Representative screenshot showing chromatin immunoprecipitation (ChIP) seq signals of H3K27ac, H3K4me2, H3K4me3, and SMARCB1 near the IL6 gene in human BJ fibroblast. (**d**) A schematic diagram of IL6 DNA regulatory regions. (**e**) ChIP showing SMARCB1 and H3K4me3 binding at the IL6 promoter in control and hIL1β-treated ARPE19 was analyzed by real-time PCR. We performed three independent experiments and analyzed statistically (mean +/− S.E.M, Statistical significance * *p* < 0.05, *** *p* < 0.001 vs. Control). (**f**) mRNA level of SMARCB1 and IL6 determined by real-time PCR in SMARCB1 overexpression and/or hIL1β-treated ARPE19. We performed three independent experiments and analyzed statistically (mean +/− S.E.M, ns: non significant, Statistical significance ** *p* < 0.01, *** *p* < 0.001 vs. Control). (**g**) Protein level of IL6 determined by ELISA in SMARCB1 knockdown and/or hIL1β-treated ARPE19 (left) and in SMARCB1 overexpression and/or hIL1β-treated ARPE19 (right). We performed three independent experiments and analyzed statistically (mean +/− S.E.M, Statistical significance ** *p* < 0.01, *** *p* < 0.001 vs. Control). (**h**) Heatmap showing expression of TNFα_signaling_via_NF-κB and IL6_JAK_STAT3 signaling genes in SMARCB1 knockdown ARPE19 and IMR90.

We confirmed the upregulation of IL6 in both SMARCB1-knockdown cell lines by RT-PCR (Figure 4a). We compared the absolute level of IL6 with mouse immune cells, which are dendritic cells (DC) and bone marrow-derived macrophages (BMM), with species-specific RT-primers. Although the levels of IL6 in ARPE19 and IMR90 cells were low, the upregulated IL6 level upon SMARCB1 knockdown was comparable to the IL6 level of immune cell DC or BMM (Figure S1a). Next, we compared the increase in IL6 by SMARCB1 knockdown with the increase induced by a well-known IL6 activator, IL1β [39]. We observed that SMARCB1 knockdown-induced upregulation of IL6 was further enhanced in the presence of human IL1β (hIL1β) (Figure 4b), suggesting that SMARCB1 was in the mainstream of IL6 regulation. To investigate how SMARCB1 regulated the level of IL6 expression, we examined whether SMARCB1 bound to the DNA regulatory region of known IL6 upstream regulators and whether the gene expression was altered according to the presence or absence of SMARCB1 [40,41,42]. While the transcription level of these genes did not significantly change in our system (Figure S1b), we found that SMARCB1 bound to the regulatory region of activator nuclear factor-κB subunit (NF-κB) 1, repressor tumor protein p53 (TP53), stabilizer AT-rich interactive domain-containing protein 5A (ARIDA5), and degrader ZC2H12A in human BJ fibroblasts (Figure S1c). Of note, we found a significant SMARCB1 peak around the IL6 promoter in human fibroblast (Figure 4c), and it was further confirmed by chromatin immunoprecipitation (ChIP) PCR that a decrease in SMARCB1 binding and an increase in active histone marker H3K4me3 occurred simultaneously with SMARCB1 loss in ARPE19 (Figure 4d,e), suggesting that SMARCB1 inhibited IL6 transcription when cells were in a steady state, thereby reducing the unnecessary immune response of the cell. However, after receiving the immune stimulation, SMARCB1 dissociated from the promoter of IL6 and induced an immune reaction through IL6 transcription. This was also demonstrated by a gain-of-function study showing that ectopic expression of SMARCB1 induced IL6 downregulation in steady and immune-active states (Figure 4f). Interestingly, we found that hIL1β downregulates SMARCB1 expression. Although the exact mechanism cannot be described in here, hIL1β appears to down-regulate SMARCB1 transcription for efficient IL6 expression. It is worth investigating in the future. (Figure 4b,f). Next, we used enzyme-linked immunosorbent assay (ELISA) to determine the effect of SMARCB1 on IL6 secretion. Correlating with the IL6 transcription level results, IL6 production was increased by SMARCB1 knockdown and synergistically increased with hIL1β treatment (Figure 4g, left). By contrast, IL6 production was decreased by SMARCB1 overexpression, and the effects of hIL1β treatment were halved (Figure 4g, right). These data indicated that SMARCB1 regulated not only IL6 transcription but also IL6 secretion. Furthermore, these effects led to IL6 downstream signaling (Figure 4h). This implied that SMARCB1 might be a modulator that effectively inhibits IL6 to prevent unnecessary immune responses.

## 3. Discussion

This study revealed the role of SMARCB1 in the normal cell state. SMARCB1 controls the cell cycle through p21 regulation, as well as cell maintenance-associated biological functions. These functions could explain the resultant modulation of the cell cycle following SMARCB1 knockdown. Transcriptome analysis suggested that SMARCB1 was deeply involved in inflammation-associated genes, and subsequent experiments further revealed an underlying molecular mechanism of SMARCB1 binding to the IL6 promoter to repress its expression and secretion. These results provide insight into the role of SMARCB1 as a gatekeeper of immune function in a steady cell context.

IL6 is a multifunctional cytokine that performs diverse functions regulating the overall immune system [43,44]. IL6 is produced rapidly and transiently in damaged tissue to stimulate the acute phase response and immune reactions [42]. According to Tanaka et al., there are four groups that regulate IL6. Each group of proteins promotes or represses IL6 transcription or stabilizes or degrades IL6 protein. The transcription promotion group contains NF-κB, and the transcriptional repression group contains p53 [42]. Although SMARCB1 binds to the regulatory region of NF-κB, its expression level remains unchanged following SMARCB1 knockdown, and this phenomenon was also observed in other IL6 upstream regulators. Thus, we expected that SMARCB1 would be able to modulate IL6 in multiple directions, either transcriptionally or post-transcriptionally, but we have yet to identify the exact mechanism between SMARCB1 and upstream regulators. Further investigation of their mechanism and correlation will be an interesting follow-up study on IL6. On the other hand, IL6 is known to be regulated by epigenetic mechanisms, such as DNA methylation and histone modifications, and the active transcription of IL6 is usually correlated with DNA hypomethylation and active histone modifications in several contexts [45,46]. We also showed an increase in H3K4me3 at the IL6 promoter in the presence of hIL1β with decreased SMARCB1 binding (Figure 4e). Further investigation is necessary to elucidate the underlying mechanism and causality between the dissociation of SMARCB1 and the enrichment of active histone modification upon hIL1β treatment.

SMARCB1 has been known to regulate the cell cycle through P21 in malignant rhabdoid tumors (MRT), which are the prototypical SMARCB1-deficient tumors [47]. We also found that SMARCB1 knockdown induces cell cycle arrest with P21 upregulation in non-cancer cell lines ARPE19 and IMR90 (Figure 1e,f), suggesting that P21 could be a universal target of SMARCB1 to regulate the cell cycle. Normal cells have their own immune system for protection, and those that make up the first line of defense show physical and chemical characteristics specific to immune defense [48,49,50]. ARPE19 eliminates invading pathogens to protect the eye [51], and IMR90 produces cytokines and chemokines [52]. Therefore, ARPE19 and IMR90 can be considered first-line defense cells. Considering the origin of ARPE19 and IMR90, regulation of the immune response by SMARCB1 may be relevant and better observed than in other cells of other origins, but we believe that this phenomenon can be expanded in other contexts, especially SMARCB1-dysregulated cells, such as those in cancers. Furthermore, inflammation is deeply associated with cancer progression. More recently, chronic inflammation has been considered a characteristic of advanced cancer [53], and immunotherapy is thought to be an innovative cancer treatment [54,55]. Overall, we suggest that future studies of the role of SMARCB1 in immunity be conducted considering both the functions of an immunostimulant and an immune suppressor in normal and cancer cells.

Accumulative research has established several roles of the SWI/SNF complex in physiological and pathological processes [56]. Notably, approximately 20% of human cancers are mutated in the SWI/SNF complex [57]. Therefore, studies that identify the SWI/SNF molecular network are inevitable. Our study has established a new molecular link between SMARCB1 and IL6 in the immune reaction and highlights SMARCB1 as a key molecule in the development of treatment for inflammation-related diseases, including cancers.

## 4. Materials and Methods

### 4.1. Cell Culture

The human immortalized cell lines ARPE19 and IMR90 were purchased from ATCC. Each cell line was maintained in DMEM (Welgene, Korea) and DEME/F12 (Gibco, Korea) supplemented with 10% fetal bovine serum (FBS; welgene) and 100 units/mL of penicillin–streptomycin (Invitrogen, Carlsbad, CA). All cells were cultured at 37 °C in a humidified incubator with 5% CO_2_.

### 4.2. shRNA Infection

shSMARCB1 constructs were purchased from Sigma-Aldrich. For lentivirus production, MISSION lentiviral packaging mix was used. Infected derivative cells stably expressing shRNA were selected in the presence of 1.25 μg/mL puromycin.

### 4.3. RNA Extraction and Reverse Transcription PCR

Total RNA was extracted using TRIzol reagent, digested with DNase I, and reverse-transcribed using a High-Capacity cDNA Reverse Transcription Kit (Applied Biosystems, Foster City, CA, USA). Amplification of cDNA was performed on a LightCycler^®^ 480II (Roche, Basel, Switzerland) using the LightCycler^®^ 480 SYBR Green I Master (Roche), according to the recommended conditions. cDNAs were amplified using the following gene-specific primers. Primers used were as follows: GAPDH-F: 5′-GAG TCA ACG GAT TTG GTC GT-3′, GAPDH-R: 5′-TGG AAG ATG GTG ATG GGA TT -3′, SMARCB1-F: 5′-GCT ACC ACC ATC GCA TAC-3′, SMARCB1-R: 5′-CTC CAT CTC AGC GTC TGT-3′, SMARCA4-F: 5′-GGA AGT GGC AGC GAA GAA GAC-3′, SMARCA4-R: 5′-GGG CCA GTC ACA AAC AGT CCT-3′, SMARCA2-F: CAT CTT TGA CAG CGA CTG GA-3′, SMARCA2-R: 5′-TCT GAT CCA CGT TCA GCT TG-3′, P21-F: 5′-GAG GCC GGG ATG AGT TGG GAG GAG-3′, P21-R: 5′-CAG CCG GCG TTT GGA GTG GTA GAA-3′, IL6-F: 5′-GGT ACA TCC TCG ACG GCA TCT-3′, IL6-R: 5′-GTG CCT CTT TGC TGC TTT CAC-3′. Each PCR was replicated three times and then statistically analyzed.

### 4.4. Chromatin Immunoprecipitation Assay (ChIP)

ChIP assays were performed according to instructions from Upstate Biotechnology (Lake Placid, NY). For each assay, 50 μg DNA, sheared by a sonication (the DNA fragment size was 200 to 500 bp), was pre-cleared with protein A magnetic beads (Upstate Biotechnology, cat. #16–661) and then 50 μg DNA was precipitated by SMARCB1 (Cambridge, United Kingdom, abcam #12167) and H3K4me3 (active motif #39159). After immunoprecipitation (IP), recovered chromatin fragments were subjected to real-time PCR. IgG control experiments were performed for all ChIPs and incorporated into the IP/Input (1%) by presenting the results as (IP-IgG)/(Input-IgG). ChIP Primers used were as follows: IL6_Promoter-F: TCG TGC ATG ACT TCA GCT TT-3′, IL6_Promoter-R: 5′- TGT GAC GTC CTT TAG CAT GG-3′. Each ChIP PCR was replicated three times and then statistically analyzed.

### 4.5. ChIP Sequencing

This data set was obtained from the National Center for Biotechnology Information (NCBI) Gene Expression Omnibus (GEO) database (Accession No. GSE128327).

### 4.6. Transcriptome Analysis

Biotinylated cRNA were prepared from 0.55 μg total RNA using the Illumina TotalPrep RNA Amplification Kit (Ambion, Austin, TX, USA). Following fragmentation, 0.75 μg of cRNA was hybridized to the Illumina Expression Beadchip (Illumina HumanHT-12 v4 Expression BeadChip (Illumina, Inc., San Diego, CA)) according to the protocols provided by the manufacturer. Arrays were scanned using the Illumina Bead Array Reader Confocal Scanner. Array data export processing and analysis was performed using Illumina GenomeStudio v2011.1 (Gene Expression Module v1.9.0). Array probes were logarithm-transformed and normalized by the quantile method. Each sample was duplicated. This data set was obtained from the National Center for Biotechnology Information (NCBI) Gene Expression Omnibus (GEO) database (Accession No. GSE120831).

### 4.7. IPA (Ingenuity^®^ Pathway Analysis)

IPA is a web-based software application for the analysis, integration, and interpretation of data derived from ‘omics experiments, such as RNAseq, small RNAseq, microarrays including miRNA and SNP, metabolomics, proteomics, and small-scale experiments that generate gene and chemical lists. Powerful analysis and search tools uncover the significance of data and identify new targets or candidate biomarkers within the context of biological systems. (http://www.ingenuity.com/products/ipa).

### 4.8. GSEA (Gene Set Enrichment Analysis)

GSEA was performed using the GSEA v4.0.3 software. All gene set files for this analysis were obtained from the GSEA website (www.broadinstitute.org/gsea/). An enrichment map was used for visualization of the GSEA results. An enrichment score (ES) and false discovery rate (FDR) value were applied to overlook potentially significant results.

### 4.9. Gene Ontology Analysis

Functional enrichment analysis of DEGs was performed using DAVID online tools (version DAVID 6.8; http://david.ncifcrf.gov/). The GO terms were classified into three categories: Biological process (BP); cellular component (CC); and molecular function (MF). The upregulated DEGs and downregulated DEGs were entered separately and *p* < 0.01 was considered to indicate a statistically significant difference.

### 4.10. IL6 ELISA

IL6 collected from culture media were measured in duplicate using a commercially available human IL6 quantikine ELISA kit (BD #555220) according to the manufacturer’s instructions. The antibodies used in IL6 determination do not cross-react with other cytokines. The limit of detection was 0.70 pg/mL for IL6, respectively. Both the intra- and inter-assay coefficients of variation were less than 10%.

### 4.11. Cell Cycle Analysis

After SMARCB1 knockdown with the indicated condition, cells were collected by trypsinization and cell cycle assays were performed using the Cycle test Plus DNA Reagent Kit (Seoul, Korea, BD Biosciences), according to the manufacturer’s instructions. The profiles of cells in the cell cycle were analyzed using a FACS can flow cytometer (BD Biosciences).

### 4.12. Western Blot Analysis

Cells were lysed with RIPA (Radioimmunoprecipitation assay) buffer and sonicated briefly. Cell lysates were boiled in Laemmli sample buffer, and 30 μg of each protein was subjected to SDS-PAGE. The protein concentration was measured by Bradford protein assay. Antibodies against SMARCB1 (abcam #ab12167) and BETA-ACTIN (CST #4967S) were purchased from the indicated companies.

### 4.13. MTT (3-(4,5-Dimethylthiazol-2-yl)-2,5-diphenyltetrazolium Bromide) Assay

The viability of cells after SMARCB1 knockdown was determined using the MTT assay kit (abcam, #ab211091), according to the manufacturer’s instructions. The absorbance was recorded on a microplate reader at a wavelength of 570 nm.

### 4.14. Western Blot Quantitation

Program “Image J” was used to quantify Western blot.

### 4.15. Statistical Analyses

Results are expressed as mean ± SEM. For statistical comparisons, one-way ANOVA was used, followed by Tukey’s HSD (honest significance) post hoc tests for multiple comparison using program “prism.” A *p* value <0.05 was considered significant.

## Supplementary Materials

Supplementary Materials can be found at https://www.mdpi.com/1422-0067/21/11/3969/s1, Figure S1. SMARCB1 binds to regulatory region of IL6 upstream regulators without transcription level change.

## Abbreviations

SMARC	SWI/SNF-related matrix-associated actin-dependent regulator of chromatin
SWI/SNF	switch/sucrose non-fermentable
IL	interleukin
ATP	adenosine triphosphate
BRM	Brahma homolog
BRG1	brahma-related gene 1
BAF	BRG1-associated factor
RB	retinoblastoma
E2F	E2 promoter binding factor
OCT4	octamer-binding transcription factor 4
RPE	retinal pigment epithelium
H-Tert	Human telomerase reverse transcriptase
PBRM1	Protein polybromo 1
MTT	3-(4,5-dimethylthiazol-2-yl)-2,5-diphenyltetrazolium bromide
Th2	T-helper 2
IFN	interferon
sh	short hairpin
RT	reverse transcription
PCR	polymerase chain reaction
IPA	ingenuity pathway analysis
GSEA	gene set enrichment analysis
TNF	Tumor necrosis factor
MAPK	mitogen activated protein kinase
DC	Dendritic cells
BMM	Bone marrow derived macrophages
hIL1β	human IL1β
NF-κB	nuclear factor-κB subunit
ARID5A	AT-rich interactive domain-containing protein 5A
TP53	tumor protein p53
ChIP	chromatin immunoprecipitation
ELISA	enzyme-linked immunosorbent assay
MRT	Malignant rhabdoid tumor
