# Supplementary material for: SMARCB1 Acts as a Quiescent Gatekeeper for Cell Cycle and Immune Response in Human Cells
Source: Int J Mol Sci. 2020 Jun 1;21(11):3969. doi: 10.3390/ijms21113969 (PMC7312701; doi:10.3390/ijms21113969)
Supplement: Supplementary file 1 [file ijms-21-03969-s001.pdf]

S.1. Supplementary data

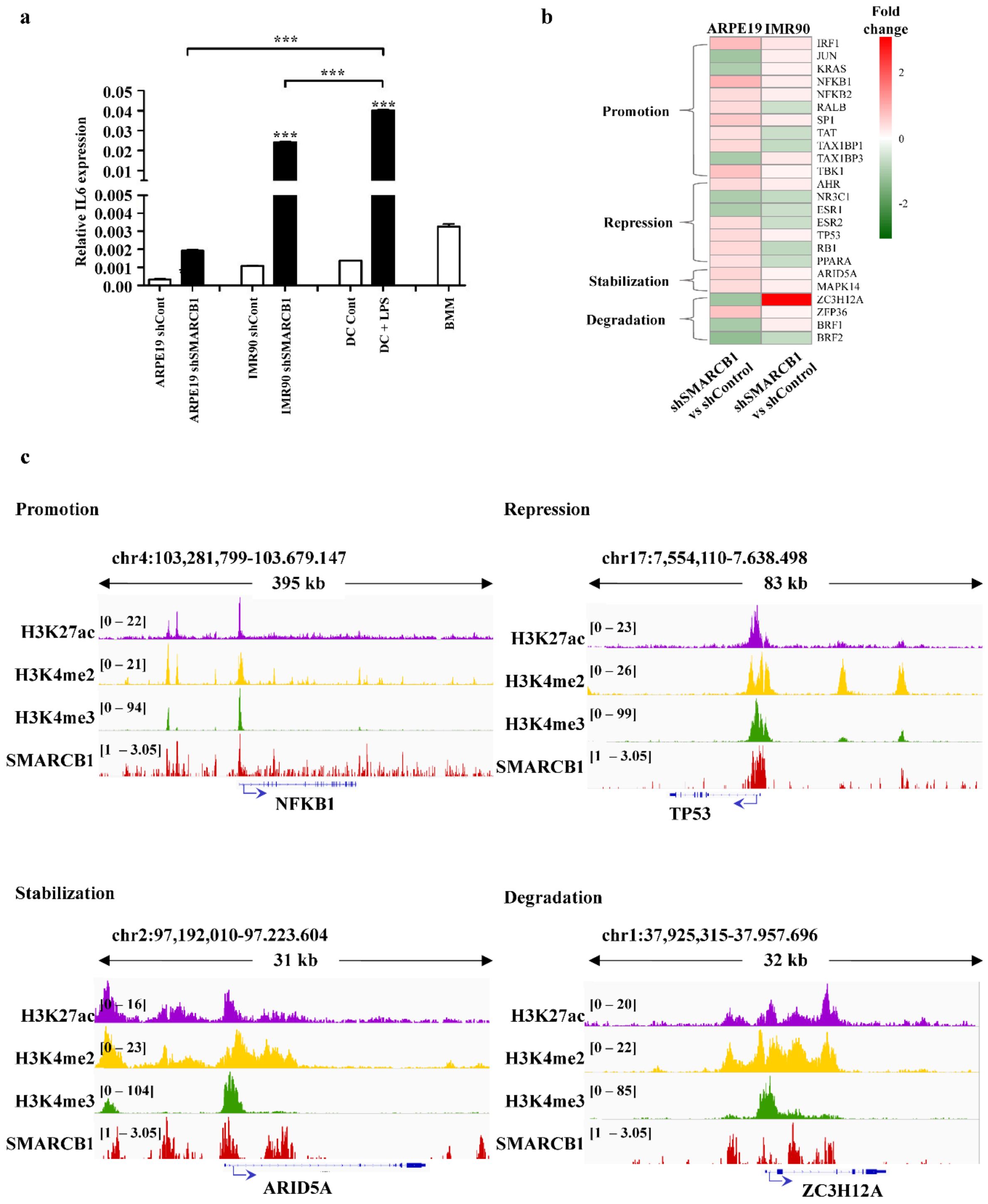

Figure S1. SMARCB1 binds to regulatory region of IL6 upstream regulators without transcription level change. (**a**) mRNA level of IL6 determined by Real time PCR. We performed three independent experiments and analyzed statistically (mean +/- S.E.M, Statistical significance ***$p < 0.001$ vs control). (**b**) Heatmap showing expression of IL6 upstream regulators in SMARCB1 knockdown ARPE19 and IMR90. (**c**) Representative screenshot showing ChIP seq signals of H3K27ac, H3K4me2, H3K4me3 and SMARCB1 near the IL6 promotion, repression, stabilization, and degradation gene in human BJ fibroblast.
